# The effect of foetal growth restriction on the development of migraine and tension-type headache in adulthood. The HUNT Study

**DOI:** 10.1371/journal.pone.0175908

**Published:** 2017-04-14

**Authors:** Sigrid Børte, Bendik S. Winsvold, Synne Øien Stensland, Milada Cvancarova Småstuen, John-Anker Zwart

**Affiliations:** 1 Institute of Clinical Medicine, Faculty of Medicine, University of Oslo, Oslo, Norway; 2 FORMI, Oslo University Hospital, Ullevål, Oslo, Norway; 3 Department of Neurology, Oslo University Hospital, Oslo, Norway; 4 Norwegian Centre for Violence and Traumatic Stress Studies, Oslo, Norway; 5 Oslo and Akershus University College of Applied Sciences, Faculty of Health Sciences, Department of Nursing and Health Promotion, Oslo, Norway; Hopital Robert Debre, FRANCE

## Abstract

**Background:**

There is little knowledge about how factors early in life affect the development of migraine and tension-type headache. We aimed to examine whether growth restriction in utero is associated with development of migraine and frequent tension-type headache in adults.

**Methods:**

The population-based Nord-Trøndelag Health Study (HUNT 3) contained a validated headache questionnaire, which differentiated between migraine and tension-type headache. These data were linked to information on weight and gestational age at birth from the Norwegian Medical Birth Registry. In total 4557 females and 2789 males, aged 19–41 years, were included in this registry-based study. Participants were categorized as appropriate for gestational age (AGA, 10^th^-90^th^ percentile), small for gestational age (SGA, 3^rd^-10^th^ percentile) or very small for gestational age (VSGA, < 3^rd^ percentile). Logistic regression was used to calculate odds ratios (OR) with 95% confidence intervals (CI) for migraine and tension-type headache, with exposure being growth restriction at birth.

**Results:**

The effect of growth restriction on migraine was modified by sex, with a significant association in males (p<0.001), but not in females (p = 0.20). In particular, males born VSGA were at increased risk of developing migraine (OR 2.73, 95% CI 1.63–4.58, p<0.001), with an intermediate risk among those born SGA (OR 1.50, 95% CI 0.96–2.35, p = 0.08) compared to those born AGA. There was no significant association between growth restriction and frequent TTH (p = 0.051).

**Conclusion:**

Growth restriction was associated with increased risk of migraine in adulthood among males, but not among females. This suggests that migraine might, in part, be influenced by early life events, and that males seem to be particularly vulnerable.

## Introduction

Migraine and tension-type headache (TTH) are the most common primary headache disorders, affecting about 15% and 21% of the world’s population respectively [1, 2]. Headache disorders have a high cost to the society because they tend to affect people during their most productive years [1], and migraine alone is ranked as one of the top ten causes of disability worldwide [3]. For both migraine and TTH, genetic variation is estimated to account for 40–50% of the risk [4]. Various lifestyle and psychosocial factors are in different studies found to be associated with headache [5–10] and for migraine, female sex hormones are suggested to play a role [11, 12].

Foetal growth restriction (FGR) is traditionally defined as birth weight by gestational age and gender below the 10^th^ percentile. FGR affects up to 9% of pregnancies in developed countries, and six times that number in developing countries [13]. Causes of FGR include chromosomal abnormalities, infection, multiples, maternal malnutrition and lifestyle factors, and placental factors. Placental insufficiency is recognized as the most important end determinant of FGR [13]. FGR is associated with both short- and long-term neurodevelopmental delays and cognitive dysfunction [13], as well as with late-onset disorders such as type 2 diabetes and cardiovascular disease [14, 15].

Few previous studies have examined the effect of pre- and perinatal factors on the risk of developing headache later in life, and no study has examined the effect of FGR on the development of headache in adulthood. In one study examining FGR and headache among 11-year old children, no effect was found on FGR and migraine, but the sample included an age group where migraine has low prevalence [16]. Only a few studies have examined other perinatal factors and headache [17–19]. Our aim was to examine a possible association between FGR and headache in adulthood.

We utilized the population-based Nord-Trøndelag Health Survey (HUNT), in which participants were assessed for headache disorders. In order to examine the effect of FGR on the development of migraine and TTH in adulthood, survey data were linked to the Norwegian Medical Birth Registry for near complete objective body measurements at birth.

## Materials and methods

### Study sample

All inhabitants 20 years or older in Nord-Trøndelag county of Norway were invited to participate in the Nord-Trøndelag Health Study, HUNT 3 (2006–2008). The study population, including both participants and non-participants has been described in detail previously [20, 21]. In brief, two questionnaires including more than 200 health-related questions were given to the participants. Of the 94 194 individuals invited, 50 839 (54%) answered the first questionnaire (Q1) which was enclosed with the invitation letter. They were also invited to participate in a brief medical examination, and to fill in a second questionnaire (Q2), which included a total of 14 headache questions. In total, 39 701 (42%) participants answered Q2 and could be classified according to headache status.

Information on weight by gestational age was obtain through The Norwegian Medical Birth Registry, which contains information on all registered births in Norway since 1967 [22]. Of those answering the headache questions, 8380 individuals were born in 1967 or later, and were therefore eligible for inclusion in the study. Of these, 7763 (92.6%) had complete information about sex, age, weight and gestational age in the registry. We subsequently excluded those who were registered with congenital malformations (n = 199), had been born < 20^th^ or > 44^th^ weeks of gestational age (n = 69), had weight for gestational age > 5 standard deviations (SD) away from the Scandinavian reference mean [23] (n = 14), were part of multiple births (n = 111) or whose mothers had rubella during the pregnancy (n = 16). Both congenital malformations, maternal rubella infection during pregnancy and multiple births are well-known causes of low birth weight [24], and therefore these individuals were excluded to avoid a potential confounding effect. After exclusions, 7354 individuals, with a mean age of 32.2 years (range from 19.2 to 41.4 years) were included in the study. For analyses of specific headache diagnoses, only those who could be classified as having migraine, frequent TTH or as headache free were included (n = 6218, Table 1).

**Table 1:** Characteristics of the Participants.

	No headache		Migraine		Frequent TTH	
Characteristics	n = 3723		n = 1335		n = 1160	
	Females	Males	Females	Males	Females	Males
Participants (n)	1974	1749	1041	294	803	357
Age, years (SD)	31.6 (6.4)	32.3 (6.2)	32.4 (6.0)**	33.1 (5.9)	32.3 (6.3)*	33.5 (5.8)**
Birth weight, grams (SD)	3492 (512)	3641 (543)	3454 (515)	3570 (541)*	3452 (529)	3650 (513)
Gestational age, weeks (SD)	40.1 (1.7)	39.9 (1.8)	40.0 (1.8)	39.8 (1.7)	40.0 (1.7)	40.0 (1.8)
Birth weight for gestational age						
AGA, % (n)	79.3 (1565)	80.8 (1413)	79.4 (827)	74.5 (219)	77.1 (619)	81.5 (291)
SGA, % (n)	6.9 (137)	6.4 (112)	8.3 (86)	8.8 (26)	9.6 (77)*	5.3 (19)
VSGA, % (n)	3.9 (76)	3.0 (52)	4.5 (47)	7.5 (22)***	5.0 (40)	3.4 (12)
LGA, % (n)	9.9 (196)	9.8 (172)	7.8 (81)	9.2 (27)	8.3 (67)	9.8 (35)

TTH: Tension-type headache; n: Numbers of participants, SD: Standard Deviation; AGA: Appropriate for gestational age (weight by percentile); VSGA: Very small for gestational age (< 3^rd^ percentile); LGA: Large for gestational age (>90^th^ percentile). Group mean or proportion is significantly different than no-headache control mean or proportion for the same sex at *p < 0.05 ** p < 0.01 *** p < 0.001 as determined by chi-square tests for categorical variables and independent t-tests for continuous variables.

Participation was based on informed, written consent, and the study was approved by the Regional Committee for Medical and Health Research (2015/463/REK midt).

### Headache diagnoses

The 14 headache questions in the second questionnaire (Q2) were designed mainly to determine whether the individual had headache, the frequency of headache, and, when headache were reported, to diagnose migraine and TTH according to a modified version of the second version of the International Classification of Headache Disorders (ICHD-II) [25]. Subjects who answered “yes” to the screening question “Have you suffered from headache during the last 12 months?” were classified as headache sufferers. Those who answered “no” comprise the headache-free control group. Based on subsequent headache questions [20], headache sufferers were classified as having migraine if they fulfilled the following four criteria: (i) Headache attacks lasting ≤ 72 hours. (ii) Headache with at least two of the following characteristics: pulsating quality, unilateral location, moderate to severe intensity or aggravation by physical activity. (iii) During headache, at least one of the following: a) nausea and/or vomiting, b) photophobia and phonophobia. Headache sufferers were classified as having frequent TTH if they fulfilled the following criteria: (i) Headache at least one day a month. (ii) Headache with at least two of the following characteristics: bilateral location, pressing quality, mild to moderate intensity and no aggravation by physical activity. (iii) During headache no nausea or vomiting and no phonophobia or photophobia. The headache diagnoses were mutually exclusive. These headache diagnoses have previously been validated against clinical interviews by neurologists [20]. The sensitivity and specificity were, respectively, 88% and 86% for any headache (Cohen’s kappa (κ) = 0.70, 95% CI 0.61–0.79), 51% and 95% for migraine (κ = 0.50, 95% CI 0.32–0.68) and 96% and 69% for frequent TTH (κ = 0.44, 95% CI 0.30–0.58).

### Foetal growth restriction (FGR)

We used the traditional definition of FGR, that is a birth weight by gestational age and gender below the 10^th^ percentile, the definition being equal to small for gestational age (SGA) [26]. Since it has been shown that this definition includes a considerable proportion of constitutionally small foetuses [27], some have suggested using a threshold of two standard deviations below the mean (corresponding to the 3^rd^ percentile) to better identify foetuses at risk [28, 29]. For the analysis in this study we therefore defined two mutually exclusive groups, those lying between the 3^rd^ and 10^th^ percentile, termed SGA, and those below the 3^rd^ percentile, termed very small for gestational age (VSGA). Participants born with a weight appropriate for gestational age (AGA, 10^th^-90^th^ percentile) were used as a reference group. Those born large for gestational age (LGA, > 90^th^ percentile) were included in prevalence calculations, but were not included in analyses of FGR. In order to calculate the percentile for each individual, birth weight was standardized according to gestational age and sex based on national reference data [23], and expressed as a z-score.

Additional information from the Norwegian Medical Birth Registry, such as specific diagnoses in the newborn, complications during pregnancy or labour, maternal smoking, alcohol use, medical conditions and medication use, could unfortunately not be included, due to lack of data or high level of missingness in the birth notification forms used before 1999.

### Statistical analysis

Considering an explanatory strategy, our major hypothesis was that FGR is associated with headache status in adulthood. We estimated the effect of having FGR at birth (exposure) on the presence of headache in HUNT 3 (outcome) using binary logistic regression. Those suffering from migraine and frequent TTH were examined separately, and compared to participants free of headache. For the exposure variable, the effect of SGA and VSGA was examined by using those born AGA as a reference. The results are reported as odds ratios (OR) with 95% confidence intervals (95% CI). The exposure variables (AGA, SGA, VSGA) were also incorporated as a single ordinal variable in a two-sided test for trend. Sex and age were considered as potential confounders or effect modifiers on this association. The confounding effect was quantified by comparing the adjusted OR to the crude OR. Variables with a confounding effect > 5% were included in the final analysis. Effect modification was examined by including an interaction term in the model, where we defined effect modification as significant interaction at p < 0.05. Modification was handled by performing stratified analyses. All tests were two-sided and p-values < 0.05 were considered statistically significant.

Estimated marginal probabilities of migraine were based on a model including the interaction of gender and growth restriction, holding age at its mean, and visualized graphically. For this analysis we included the whole study sample, including those with non-classified headache, in order to obtain representative probability estimates.

All analyses were performed using Stata/SE 14.1 for Mac (StataCorp LP, College Station, TX, USA).

## Results

### Migraine

To study the association between FGR and migraine we included 1335 subjects with migraine and 3723 subjects without headache. The overall prevalence of migraine in our study sample was 22.8% for females and 10.5% for males, which is comparable to the expected migraine prevalence for this age group, in this region [12, 30]. The mean age was 32.5 years among all the participants, with a range from 19–41 years. Characteristics of the participants are presented in Table 1. Females in the migraine group were significantly older compared to the control group, the groups were otherwise comparable. Males in the migraine group had lower birth weight than the control group, and a different distribution of weight by gestational age, with a higher proportion of individuals being VSGA compared to the control group.

There was a significant effect modification of sex on the relationship between being born VSGA and migraine (p = 0.009), and subsequent analyses were therefore performed separately for males and females. Including age in the model did not alter the estimates, and age was therefore not included as a covariate in the final model. The results are summarized in Table 2. For migraine the unstratified analyses revealed an OR of 1.27 (95% CI 0.99–1.61, p = 0.056), being SGA, and an OR of 1.52 (95% CI 1.10–2.10, p = 0.011), being VSGA, compared to those being AGA. The effect of FGR on the development of migraine was however dependent on its interaction with sex. The effect was stronger in males, for whom there was a significant association between FGR and the development of migraine (p for trend <0.001). There was a non-significantly, moderate increased odds in those born SGA (OR = 1.50, 95% CI 0.96–2.35, p = 0.08), and a significantly nearly three times higher odds of migraine among those born VSGA (OR = 2.73, 95% CI 1.63–4.58, p<0.001), compared to those born AGA. In females, no significant association was seen between FGR and the development of migraine (p for trend = 0.20), although the tendency was in the same direction as for males, with non-significantly increased odds for those born SGA (OR = 1.19, 95% CI 0.90–1.58, p = 0.23) and VSGA (OR = 1.17, 95% CI 0.81–1.70, p = 0.41).

**Table 2:** The Effect of Weight for Gestational Age on Development of Migraine.

	All		Females		Males	
	No headache	Migraine	No headache	Migraine	No headache	Migraine
		OR (95% CI)		OR (95% CI)		OR (95% CI)
	n	n	n	n	n	n
AGA		1.0 (ref)		1.0 (ref)		1.0 (ref)
	2978	1046	1565	827	1413	219
SGA		1.27 (0.99–1.61)		1.19 (0.90–1.58)		1.50 (0.96–2.35)
	249	112	137	86	112	26
VSGA		1.52 (1.10–2.10)		1.17 (0.81–1.70)		2.73 (1.63–4.58)
	128	69	76	47	52	22
p for trend		0.001		0.20		< 0.001

AGA: Appropriate for gestational age (weight by gestational age 10^th^-90^th^ percentile); SGA: Small for gestational age (3^rd^-10^th^ percentile); VSGA: Very small for gestational age (<3^rd^ percentile); OR: Odds ratio; CI: Confidence Interval; n: Number of participants

**Fig 1:**
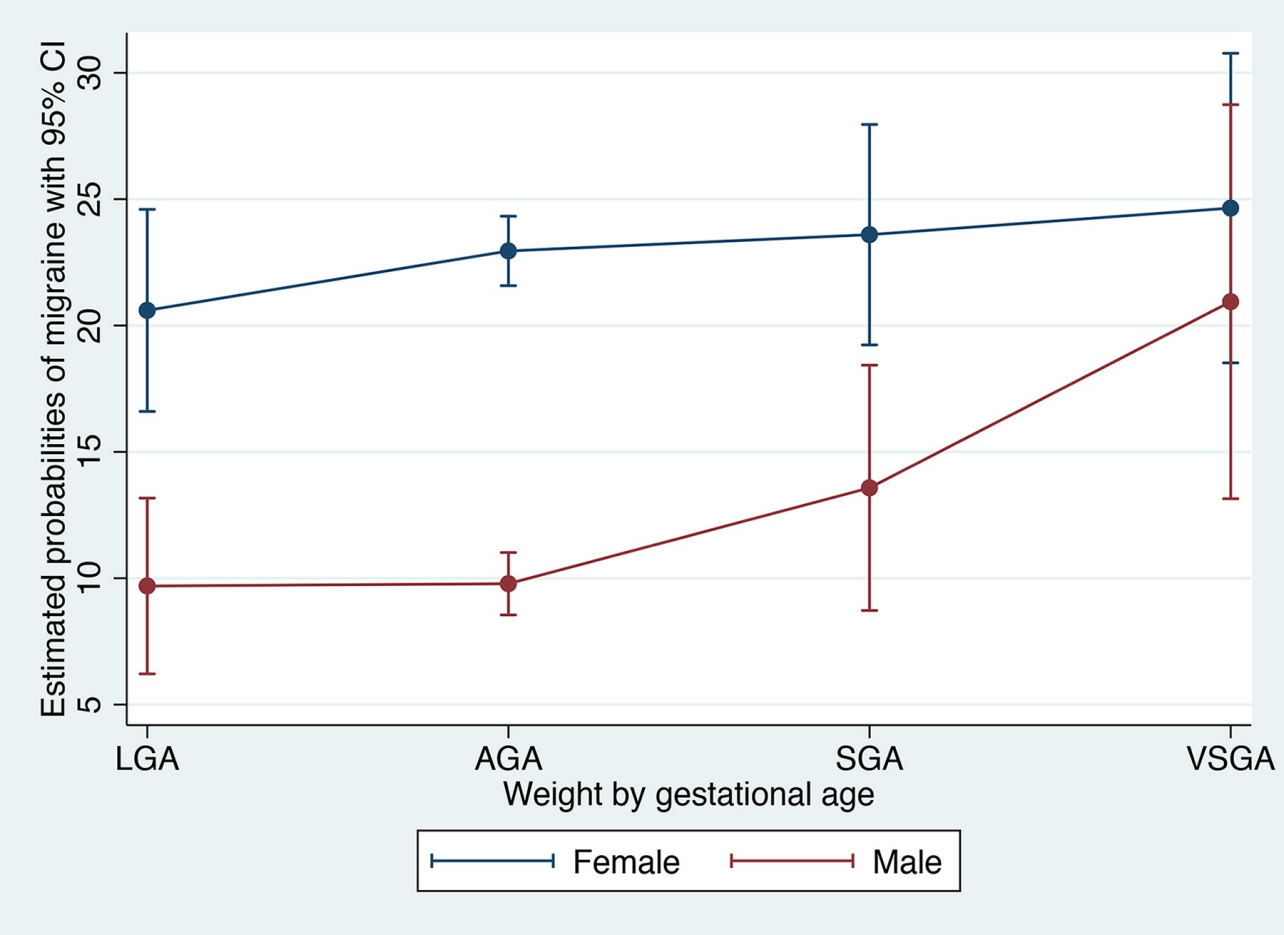
Estimated Probabilities of Migraine (%) by Weight/Gestational Age and Sex. AGA: Appropriate for gestational age (weight by gestational age 10^th^-90^th^ percentile); SGA: Small for gestational age (3^rd^-10^th^ percentile); VSGA: Very small for gestational age (<3^rd^ percentile); LGA: Large for gestational age (90^th^-100^th^ percentile).

Age-adjusted estimates for prevalence (probabilities) of migraine by sex and weight by gestational age are given in Fig 1. For males, migraine was found to be more than twice as prevalent among those born VSGA (20.9%) compared to those born AGA (9.8%).

### Frequent tension-type headache

The same procedure was used to investigate the association between FGR and frequent TTH (n = 1160). Characteristics of the participants are summarized in Table 1. Both males and females in the frequent TTH group were significantly older than the headache free control groups, and females with frequent TTH were more often born SGA.

No effect modifiers were identified. Sex had a confounding effect and was therefore included in the model, but age did not.

FGR was not significantly associated with the development of frequent TTH (p for trend = 0.051). There were no significant differences in odds for migraine for those born SGA (OR = 1.22, 95% CI 0.95–1.56, p = 0.12) or VSGA (OR = 1.27, 95% CI 0.91–1.77, p = 0.17), compared to those born AGA.

## Discussion

In this population-based study of 7354 adults with information on measurements at birth, we found that FGR was associated with increased odds of developing migraine in males. This relationship was most pronounced in those born VSGA, among whom the odds of migraine in adulthood were almost three times higher than in the non-growth restricted group. The study revealed no association between FGR and migraine in females, or between FGR and frequent TTH.

Few previous studies have investigated the effect of perinatal factors on the risk of headache. In contrast to our findings, one longitudinal study of 871 11-year old children did not report an effect of FGR on migraine or TTH [16]. The study may however have been limited by the low prevalence of migraine in this age group. As well, they included term infants only, and did not analyse females and males separately. A few additional studies have examined the impact of other perinatal factors on later headache. They have reported an association between admission to the neonatal intensive care unit and later migraine [18], and between maternal tobacco and alcohol use during pregnancy and later chronic daily headaches [17], while no association was found between being born prematurely and headache in children [19]. However, these studies were limited by small study samples or failure to separate migraine from other types of headache.

FGR is defined, by its true meaning, as a foetus who does not reach its predetermined growth potential [31]. This can be a result of genetic factors, such as chromosomal abnormalities, or of environmental factors, poor placental function being the most important contributor to FGR [13]. Insufficient placental circulation leads to chronic hypoxia and reduced nutrient supply to the foetus, resulting in a decreased growth rate and redistribution of blood to vital organs including the brain [13]. Despite this brain-sparing effect, FGR is associated with several neuropathological difficulties in childhood and later life, including impaired fine- and gross motor skills, impaired cognition and learning, and behavioural problems regarding attention, responsivity, hyperactivity, mood and anxiety [13]. FGR has been associated with attention deficit/hyperactivity disorder [32], cerebral palsy [13], and possibly with epilepsy [33].

Specific structural and functional changes in the brain associated with FGR include delayed myelination, a reduced number of synapses and altered dendritic morphology, changes in grey and white matter volume, and reduced brain connectivity particularly in the prefrontal and limbic networks, the latter correlating with neurobehavioral impairments, such as hyperactivity and cognitive executive deficits [13]. Of particular interest in relation to migraine, animal studies have shown alterations in neurotransmitter levels in various parts of the brain, with increased excitatory glutaminergic activity, and decreased inhibitory GABA-ergic activity [34], in addition to increased levels of dopamine, noradrenalin and serotonin [34–36]. Several lines of evidence suggest an imbalance of synaptic excitation-inhibition in migraine, resulting in a hyperexcitable state. This presents a potential mechanism for an increased propensity for developing migraine due to FGR [37, 38]. It is also likely that serotonin plays a role in migraine pathogenesis, with serotonin receptor agonists (triptans) being important in the acute treatment of migraine [39]. As well there is some evidence for imbalances in dopamine and noradrenaline function in migraine patients, although their specific role in the pathogenesis remains unclear [40, 41].

Several studies have also indicated that altered hemodynamics in utero may result in dysfunctional development of the cardiovascular system, “programming” the foetus for lifelong cardiovascular morbidity [42]. A higher prevalence of cardiomyopathy-like remodelling and vascular dysfunction, increased blood pressure and increased carotid intima-media thickening have been found in older children born with FGR [15]. Several lines of evidence point towards an association between migraine and vascular dysfunction [43], and FGR as a shared underlying factor could theoretically present one explanation for this relationship.

It could also be speculated that the effect of FGR on the later development of migraine might be mediated through admission to the neonatal intensive care unit (NICU), which involves exposure to painful procedures and stressful events. This experience may play a role in the later development of headaches [18].

Somewhat surprisingly, we found a significant effect modification of sex. The risk of developing migraine was clearly increased in growth restricted males, but not in females. This greater effect of FGR on males is in line with previous studies on other conditions, suggesting that it may have a biological basis [44, 45]. Compared to females, growth restricted males are at increased risk of neonatal complications and mortality [46, 47], cerebral palsy, mental- and psychomotor developmental delays, and neurodevelopmental impairment [48]. It has been suggested that male and female foetuses adapt differently to adverse environments, most likely mediated by sex specific adaptations of the foetal-placental unit regarding immune function and response to maternal inflammatory status. The female placenta seems to be more responsive to changes in glucocorticoid concentration, and adapts to an adverse intrauterine environment with altered gene- and protein expression and a minor decrease in growth of the female foetus, without it becoming growth restricted. The male placenta is more resistant to glucocorticoids and exhibits minimal adjustments, with the male foetus continuing to grow normally in the presence of an adverse intrauterine environment. In the presence of a second stressful event, the male response is associated with increased risk of growth restriction, preterm delivery or death in utero, while the female response seems to ensure survival [49]. Likewise, female foetuses appear to respond more adequately to asphyxia, with a higher release of catecholamines and a lower risk of neurological complications [47]. Lastly, there seem to be microvascular differences between male and female neonates born preterm or from pre-eclamptic pregnancies, with a stronger vasodilation at birth in males than in females [49]. These findings indicate that males are especially vulnerable to stressful perinatal events, and that reduced nutrient supply and hypoxia in utero might have greater consequences for the growing brain in males than in females. Similar mechanisms could be involved in the stronger effect of FGR on migraine in males than in females observed in our study.

We did not find any associations between being growth restricted at birth and frequent TTH. A reason for this might, in part, be that migraine and TTH are caused by separate underlying mechanisms, as has been suggested previously [50].

Strengths of our study include the large and unselected population, the use of validated headache diagnoses, and near complete objective measurements of height and weight at delivery, making it possible to define FGR.

One limitation of our study may be the low number of growth restricted headache sufferers, in particular growth restricted males with headache. However, despite large confidence intervals, the findings were still significant. Another limitation is that headache diagnoses were based on responses to a questionnaire, and not on clinical diagnosis. For this reason people who suffer from aura without headache will also not be included. Also, while we used a traditional definition of FGR, true FGR should optimally be determined using Doppler ultrasound of the foetal blood vessels during pregnancy. In particular, an abnormal ratio between cerebral and placental vascular function is a stronger predictor of pregnancy complications than birth weight by gestational age [51]. It might also have been more optimal to use customized growth charts, but unfortunately information on maternal factors like ethnicity, height, weight and age were not available. It is likely that a proportion of those defined as having FGR in the current study are simply constitutionally small [52], and not at increased risk of adverse outcomes [53]. Likewise, we will have missed growth restricted foetuses with a birth weight above the 10^th^ percentile. Both types of misclassification will result in an underestimation of the effect of FGR on the development of headache in the current study. To minimize the risk of misclassification, we studied the group born VSGA, where the proportion of true FGR will be higher, separately. Lastly, while we examined age and sex as potential confounders, we cannot exclude residual confounding from variables not available in our material, such as maternal psychiatric comorbidity, health, lifestyle and medication use during pregnancy, complications in pregnancy, complications/conditions in the newborn, socioeconomic status and other hardships that may affect later caregiving, psychosocial environment and family lifestyle. It would have been of particular value to further examine the growth restricted males with migraine, regarding the above-mentioned factors, to evaluate if they differed from the normal-weighted newborns in other ways than being small. Future studies where such factors are assessed, should consider the extent to which they affect the relationship between FGR and later migraine.

In conclusion, in this first population-based study examining the effect of FGR at birth on the later development of headache, we found that FGR was a predictor for migraine in adulthood among males. This association was strongest for those born VSGA (<3^rd^ percentile), who had almost three times higher odds of migraine in adulthood. No significant relationship between FGR and the development of migraine was found in females. That the association was limited to males corresponds with previous findings of a more prominent effect of FGR in males than in females on various neurological outcomes. No relationship was found between FGR and TTH, substantiating the notion that migraine and TTH may be influenced by different pathophysiological and psychological mechanisms. The biological mechanisms involved in a relationship between FGR and migraine remain unclear, but may include altered neurotransmitter activity or alterations in brain structure and connectivity. Further research is needed to reveal these underlying mechanisms.
